# Prediction of histopathological grade in gastric cancer using dual energy CT-derived synthetic extracellular volume fraction

**DOI:** 10.3389/fonc.2025.1688072

**Published:** 2025-12-17

**Authors:** Hai-fei Zhou, Wei Chen, Jing-qi Li, Ai-yun Sun, Li-li Guo

**Affiliations:** 1 Department of Radiology, The Affiliated Huai’an NO.1 People’s Hospital of Nanjing Medical University, Huai’an, Jiangsu, China; 2 Department of Pathology, The Affiliated Huai’an NO.1 People’s Hospital of Nanjing Medical University, Huai’an, Jiangsu, China; 3 CT Imaging Research Center, GE HealthCare China, Shanghai, China

**Keywords:** dual-energy CT, extracellular volume fraction, gastric cancer, grade of pathology, hematocrit, synthesis of ECV

## Abstract

**Purpose:**

To investigate the relationship between the synthetic extracellular volume (ECV) fraction and the hematocrit (HCT)-based ECV fraction in gastric cancer patients and to evaluate its potential utility in predicting the pathological grade of gastric cancer.

**Methods:**

Data from the derivation cohort consisting of 142 patients who underwent nonenhanced abdominal CT examination were collected, and the CT values of the abdominal aorta (CTA) and inferior vena cava (CTV) were measured separately. Using linear regression between CT attenuations and conventional hematocrit (HCT_con_), calculation formulas were derived for the synthetic hematocrit of the abdominal aorta (HCT_syn, A_) and the inferior vena cava (HCT_syn, V_). A validation cohort of 62 gastric adenocarcinoma patients with dual-energy CT was analyzed to calculate synthetic ECV (ECV_syn, A_ and ECV_syn, V_) using the derived formulas. Differences between synthetic and conventional ECV(ECVcon), and their efficacy in differentiating pathological grades, were compared.

**Results:**

Derived formulas: *HCT_syn,A%_* = 1.152×*CT_A_* - 12.311; *HCT_syn,V%_* = 1.142×*CT_V_* - 11.229. In validation, HCT_syn,A_ and HCT_syn,V_ showed no significant difference from HCT_con_ (all P>0.05). HCT_con_ was strongly correlated with HCT_syn, A_ (r = 0.898, *P* < 0.01) and HCT_syn, V_ (r = 0.826, *P* < 0.01). ECV_syn,A_ and ECV_syn,V_ also did not differ from ECV_con_ (all P>0.05). ECV_con_ was strongly correlated with ECV_syn, A_ (r = 0.990, *P* < 0.01) and ECV_syn, V_ (r = 0.983, *P* < 0.01). All ECV metrics exhibited excellent and comparable efficiency in differentiating pathological grades.

**Conclusions:**

Synthetic ECV performs comparably to conventional ECV and predicts gastric cancer pathological grading effectively.

## 1 Introduction

The extracellular volume (ECV) fraction represents the proportion of tissue volume occupied by the extracellular matrix (ECM) within the cellular microenvironment, thereby reflecting the volumetric distribution of the ECM (1). Although needle biopsy serves as the reference standard for accurately quantifying the ECV fraction, it is inherently limited to localized measurements (2). Furthermore, due to its invasive nature, needle biopsy is often poorly tolerated by patients, underscoring the clinical need for noninvasive imaging modalities in ECV assessment.

Quantification of the ECV fraction using computed tomography technology (CT-ECV) offers several advantages, including convenience, noninvasiveness, quantitative capability, and repeatability. This method holds promise as a novel imaging biomarker in clinical practice, contributing to improved understanding of disease processes and gaining increasing application in the diagnosis and prognosis of various conditions across multiple organ systems. For instance, Nacif MS et al. (3) demonstrated that the myocardial ECV fraction quantified by cardiac CT is useful for assessing myocardial fibrosis. Similarly, Bandula S (4) reported that liver ECV fractions derived from equilibrium-phase CT images correlate with histologically confirmed markers of liver fibrosis. Moreover, Fukukura et al. (5) showed that the ECV fraction can serve as a prognostic indicator in patients with stage IV unresectable pancreatic cancer undergoing chemotherapy. CT-ECV is typically quantified by assessing the distribution of contrast agents within tissues during the equilibrium phase of contrast-enhanced scanning. At this stage, the contrast agent is presumed to be in relative equilibrium between the ECM and the intravascular space. Measurements of either CT attenuations or iodine concentrations are obtained from both the region of interest (ROI) and the blood pool, while hematocrit (HCT) values obtained from routine blood tests are used to calculate the ECV calculation. However, this traditional method presents certain clinical limitations. Some patients may lack conventional HCT (HCT_con_) data, and the accuracy of ECV calculation depends on acquiring HCT_con_ values close to the time of CT scan. To address these issues, a synthetic ECV fractions method has been proposed. By establishing a regression model between the CT attenuation of a noncontrast blood pool images and the HCT values, this method enables estimation of ECV fractions even in the absence of contemporaneous blood test results.

Previous studies (6–8) have established that the synthetic ECV is strongly correlated with the HCT_con_-based ECV and is suitable for quantifying myocardial fibrosis. However, the applicability of synthetic ECV in gastrointestinal organs remains unexplored. Moreover, as the stomach is a hollow organ that undergoes continuous peristalsis, it poses additional challenges for imaging. Dual-energy computed tomography (DECT), as an advanced imaging technique, enables quantitative measurement of iodine concentration through material decomposition algorithms. Compared with conventional CT, DECT-based quantification of the ECV fraction requires only a contrast-enhanced scan during the equilibrium phase, eliminating the need for a non-contrast scan. As a result, this approach reduces radiation exposure and avoids registration errors (9, 10). There exists a certain correlation between ECV values and tumor histopathological grading. A previous study reported that ECV values were significantly higher in high-grade rectal adenocarcinoma compared with low-grade tumors, and that ECV showed a positive correlation with histopathological grade, suggesting its potential value in preoperative prediction of tumor grading (11). Therefore, the objective of this study is to investigate the correlation between synthetic ECV derived from DECT and conventional ECV measurements based on hematocrit, and to evaluate its potential clinical utility in predicting the histopathological grade of gastric cancer.

## 2 Materials and methods

### 2.1 Participants

**Figure 1:**
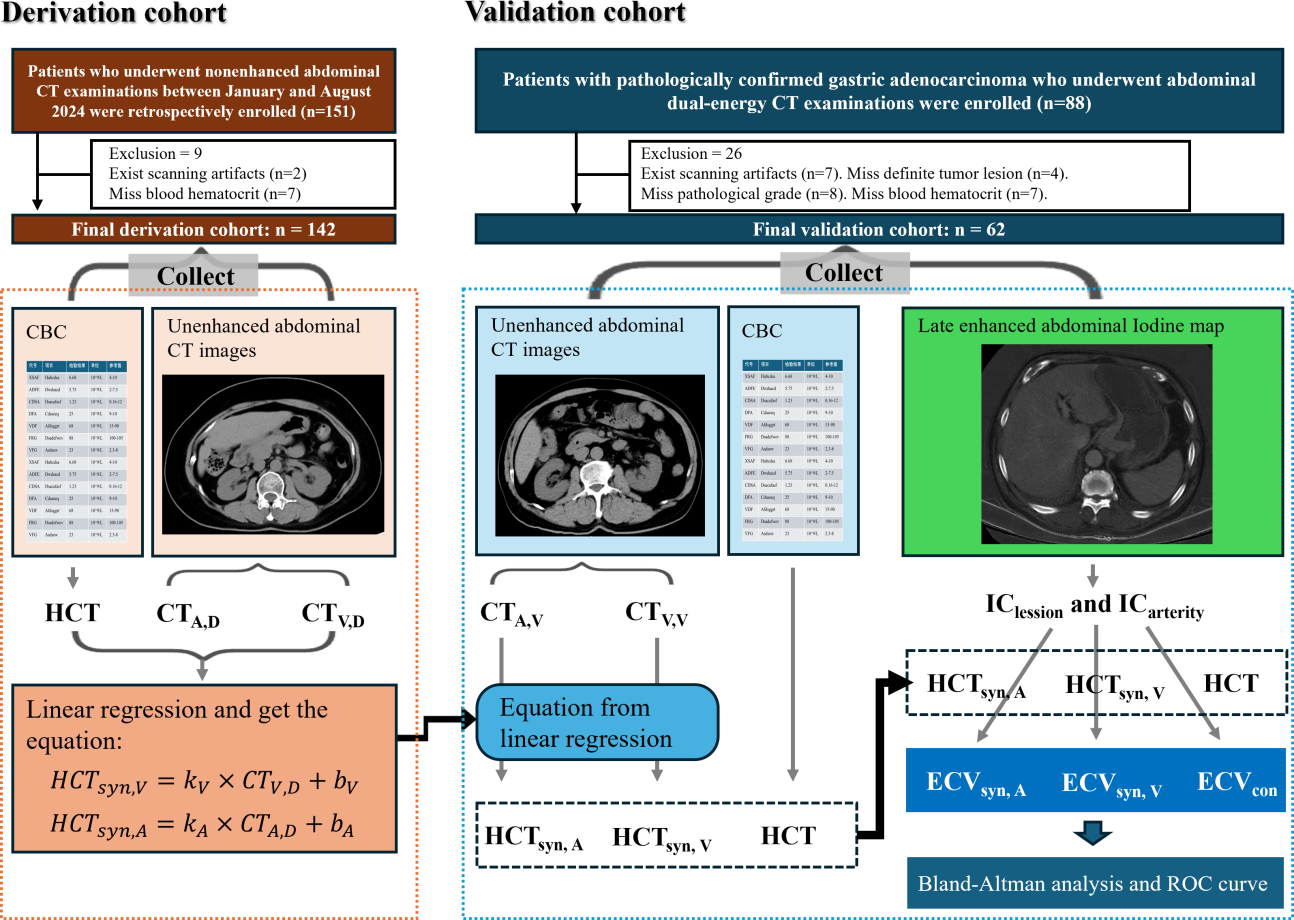
Flowchart of patient selection and synthetic ECV calculation process. CT_A, D_ and CT_V, D_ are the nonenhanced CT attenuations of the abdominal aorta and inferior vena cava in the derivation cohort, respectively. CT_A, V_ and CT_V, V_ are the nonenhanced CT attenuations of the abdominal aorta and inferior vena cava in the validation cohort. HCT_syn, A_ and HCT_syn, V_ are the synthetic HCT values derived from the CT attenuations of the abdominal aorta and inferior vena cava, respectively. HCT is the blood HCT value, ECV_syn, A_ and ECV_syn, V_ constitute the synthetic ECV calculated from synthetic HCT, and ECV_con_ is the conventional ECV. CBC, complete blood count.

This study was approved by the Ethics Committee of the Affiliated Huai’an NO.1 People’s Hospital of Nanjing Medical University (Approval No: KY-2024-135-01), and two datasets were collected in this study. The first dataset was used for linear regression to obtain the calculation formula of synthetic ECV (derivation cohort), and the second dataset was used to verify the difference between synthetic ECV and conventional ECV (HCT_con_-based ECV) and its efficacy in identifying the pathological grade of gastric cancer (validation cohort). Patients who underwent nonenhanced abdominal CT examinations between January and August 2024 were retrospectively included in the derivation cohort and informed consent was waived. The exclusion criteria were as follows: a) images with scanning artifacts; b) thrombosis in the inferior vena cava or abdominal aorta; and c) absence of HCT_con_ measurement within 24 hours before and after the scan. Patients with pathologically confirmed gastric adenocarcinoma who underwent abdominal DECT examinations were enrolled in the validation cohort and all participants provided informed consent. The exclusion criteria were as follows: a) images with scanning artifacts; b) thrombosis in the inferior vena cava or abdominal aorta; c) no definitive tumor lesion identified on CT scan; d) lack of pathological grading for gastric cancer; and e) absence of HCT_con_ measurement within 24 hours before and after the scan. **Figure 1** illustrates the design of the study.

### 2.2 Scanning protocol

All patients underwent imaging with a 256-row CT scanner (Revolution Power CT, GE Healthcare, Wisconsin, USA). For the derived cohort, an abdominal unenhanced protocol was utilized. The scanning parameters were as follows: scanning range extending from the diaphragm to the level of the pubic symphysis, matrix size of 512 × 512, tube voltage of 120 kV, tube current ranging from 150 to 650 mA, and a noise index of 8. For the validation cohort, an abdominal dual-energy protocol was employed, which required patients to fast for 12 hours prior to the examination and to orally consume 800 mL of water to distend the gastric cavity. The scanning procedure involved positioning all patients in a supine position on the scanner, followed by a conventional unenhanced CT scan with parameters identical to those used for the derivation cohort. A contrast-enhanced scan was subsequently performed in GSI mode (voltage was switched between 80/140 kVp). Other scanning parameters included detector coverage of 80 mm, tube current of 200 mA, pitch of 1.531, and rotational speed of 0.5 s. Contrast media (Ultravist370; Schering, Berlin, Germany) was administered via the median cubital vein at 2.7 mL/s using a high-pressure syringe (Ulrich REF XD 2060-Touch, Germany) at a dose of 75 mL. Following the initiation of contrast agent administration, contrast-enhanced CT images encompassing the arterial, portal venous, and equilibrium phases of the entire abdomen and pelvis were acquired. Specifically, after contrast agent administration, the arterial phase was captured at 30 seconds, the portal venous phase at 120 seconds, and the equilibrium phase at 180 seconds post-injection. Additionally, thin-slice images with a slice thickness and interval of 1.25 mm were reconstructed. To mitigate image noise, an adaptive statistical iterative reconstruction-Veo (ASiR-V) algorithm with a 40% intensity setting was applied to the reconstructed images. Iodine-based images during the equilibrium phase were generated using the GSI Volume Viewer software package on the ADW 4.7 workstation (GE Healthcare, Milwaukee, WI, USA), employing the material decomposition algorithm of spectral CT. The pixel values represent the iodine concentration (100 μg/mL).

### 2.3 Synthesis of HCT and ECV methodology

Two radiologists, each with 10 years of experience, independently measured the nonenhanced CT attenuations of the abdominal aorta (near the level of the superior mesenteric artery) and the inferior vena cava (at its maximal level) in the derivation cohort, without access to clinical data. These measurements were recorded as CT_A, D_ and CT_V, D_, respectively. Similarly, the nonenhanced CT attenuations of the abdominal aorta and inferior vena cava in the validation set were measured and recorded as CT_A, V_ and CT_V, V_, respectively, ensuring that the measurement planes were consistent with those used in the derivation cohort. Additionally, the iodine concentrations (IC) of the lesions on the iodine-based image and the aorta at the same level during the equilibrium phase in the validation cohort were measured and recorded as IC_lesion_ and IC_aorta_, respectively. Two radiologists independently drew a polygonal ROI manually outlining the largest solid portion of the tumor on the equilibrium-phase iodine-based image at the slice showing the maximum cross-sectional tumor area. Necrotic areas, intratumoral or perigastric vessels, calcifications, artifacts, and gastric lumen contents were carefully avoided. The iodine concentration of the lesion (IC_lesion_) and that of the abdominal aorta at the same level (IC_aorta_) were recorded. The aortic ROI was drawn as large as possible while avoiding the vessel wall. A third radiologist, with 5 years of experience, collected routine blood samples from all patients within 24 hours before and after the scan and recorded the venous blood HCT values. The nonenhanced CT attenuations of blood (abdominal aorta and inferior vena cava) and the HCT satisfied the following formula Equations 1, 2:

(1) $$HCT=k_{A}\times CT_{A,D}+b_{A}$$

(2) $$HCT=k_{V}\times CT_{V,D}+b_{V}$$

where *k_A_* and *b_A_* are the undetermined coefficients of HCT calculated using the unenhanced CT attenuations of the abdominal aorta in the derivation cohort and *k_V_* and *b_V_* are the undetermined coefficients of HCT calculated using the unenhanced CT attenuations of the inferior vena cava in the derivation cohort.

*k_A_*, *b_A_*, *k_V_*, and *b_V_* were determined through linear regression analysis utilizing data from the derivation cohort. Subsequently, the HCT values were computed using the nonenhanced CT attenuations of the abdominal aorta and inferior vena cava from the validation cohort, employing the following formula Equations 3, 4:

(3) $$HCT_{syn,A}=k_{A}\times CT_{A,V}+b_{A}$$

(4) $$HCT_{syn,V}=k_{V}\times CT_{V,V}+b_{V}$$

The ECV based on HCT is calculated using the following formula (2, 5) Equation 5:

(5) $$ECV=(1-HCT)\times \frac{IC_{lession}}{IC_{arteriy}}$$

**Figure 2:**
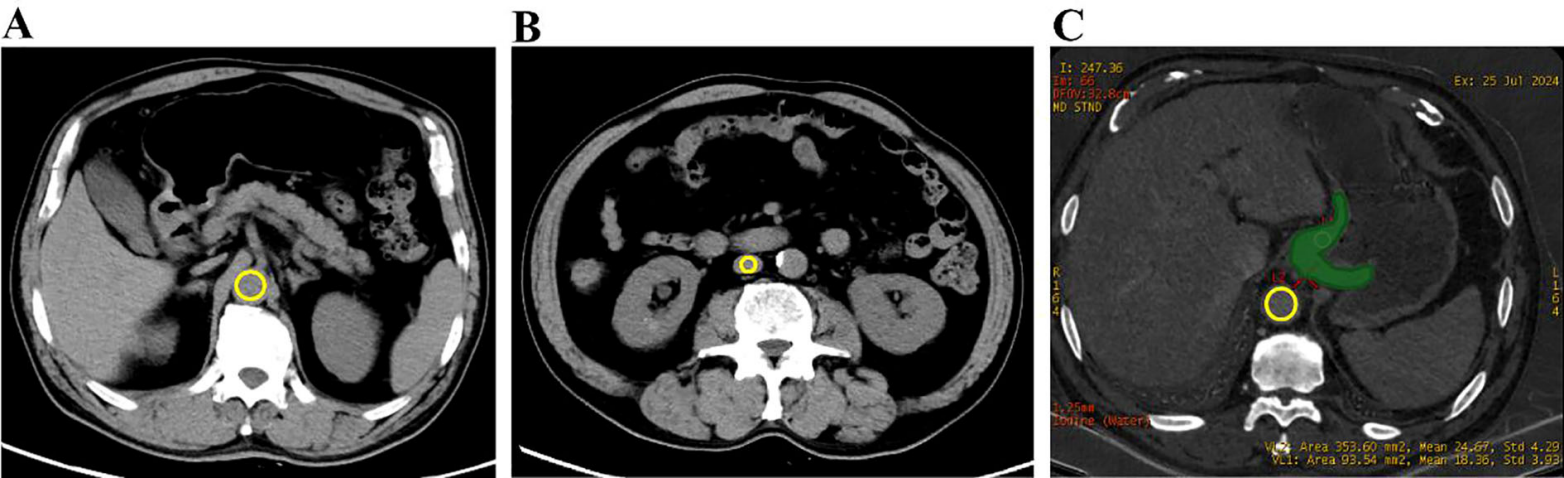
A schematic diagram of synthetic ECV measurement. **(A)** Non-contrast image, with the measured CT attenuation of the aorta being 47 HU. **(B)** Non-contrast image, with the measured CT attenuation of the inferior vena cava being 45 HU. **(C)** shows the iodine map, where the iodine value of the tumor was measured at 1.836 mg/mL (green ROI), and the iodine value of the aorta at the same level was measured at 2.467 mg/mL (yellow ROI).

The ECV based on the synthetic HCT was calculated using the following formula and **Figure 2** illustrates the schematic diagram of synthetic ECV measurement Equation 6:

(6) $$ECV_{syn}=(1-HCT_{syn})\times \frac{IC_{lession}}{IC_{arteriy}}$$

### 2.4 Pathological data analysis

A pathologist with a decade of professional experience conducted pathological image analysis based on the World Health Organization’s (WHO) fifth edition of the Gastric Cancer Pathological Diagnosis Classification (2019 edition) (12). Well-differentiated tubular adenocarcinoma was characterized by well-formed glandular structures, minimal cellular atypia, potential glandular dilation, and occasional necrosis within the glandular lumen. Moderately differentiated adenocarcinoma exhibited heterogeneous glandular morphology and intricate glandular structures, such as cribriform glands and prominent branching, budding, or fused glands. The cancer cells displayed marked atypia, with variably sized and hyperchromatic nuclei, prominent nucleoli, and frequent mitotic figures. Poorly differentiated adenocarcinoma was marked by the absence of glandular structures and the presence of scattered, linear, nested, sheet-like, or solid growth patterns of cancer cells. Based on the pathological findings, gastric cancer cases were categorized into two groups: low-grade (well-differentiated, moderately differentiated, and well-to-moderately differentiated) and high-grade (poorly differentiated and moderately-to-poorly differentiated) (13).

### 2.5 Statistical methods

Analyses were conducted utilizing IBM SPSS Statistics and MedCalc software. Data conforming to a normal distribution were reported as the means ± standard deviations, whereas data not adhering to a normal distribution were expressed as the medians and interquartile ranges. The normality of the distribution was evaluated through the Kolmogorov-Smirnov test. Comparative analyses were performed on the baseline characteristics of both the derivation and validation cohorts. A derived HCT was calculated based on the non-enhanced CT attenuations of the blood, with the model constructed via linear regression analysis, wherein the nonenhanced CT attenuation served as the independent variable and the measured HCT as the dependent variable. The intraclass correlation coefficient (ICC) was used to assess the repeatability of the measurements. *ICC* ≤ 0.40 indicated a poor consistency; 0.40 <ICC < 0.75 indicated a moderate consistency; and ICC ≥ 0.75 indicated a good consistency. The differences between synthetic HCT and conventional HCT, as well as between synthetic ECV and conventional ECV, were evaluated using either the paired *t* test or Wilcoxon signed-rank test. Correlation analyses between synthetic HCT and blood HCT and between synthetic ECV and conventional ECV were conducted using the Pearson or Spearman correlation coefficient (*r*). In the correlation test, the reference levels were defined as follows: *r* ≤ 0.40 indicated a poor correlation; 0.40 < r < 0.75 indicated a moderate correlation; and r ≥ 0.75 indicated a good correlation. The Bland-Altman method was employed to assess the agreement between conventional ECV and synthetic ECV. The differences in ECV values between the high-grade and low-grade groups, as well as the predictive values of both conventional ECV and synthetic ECV for the pathological grading of gastric cancer, were calculated. Finally, the DeLong test was used to compare the differences among the ROC curves. A *P* value < 0.05 was considered statistically significant.

## 3 Results

### 3.1 Baseline characteristics

**Figure 3:**
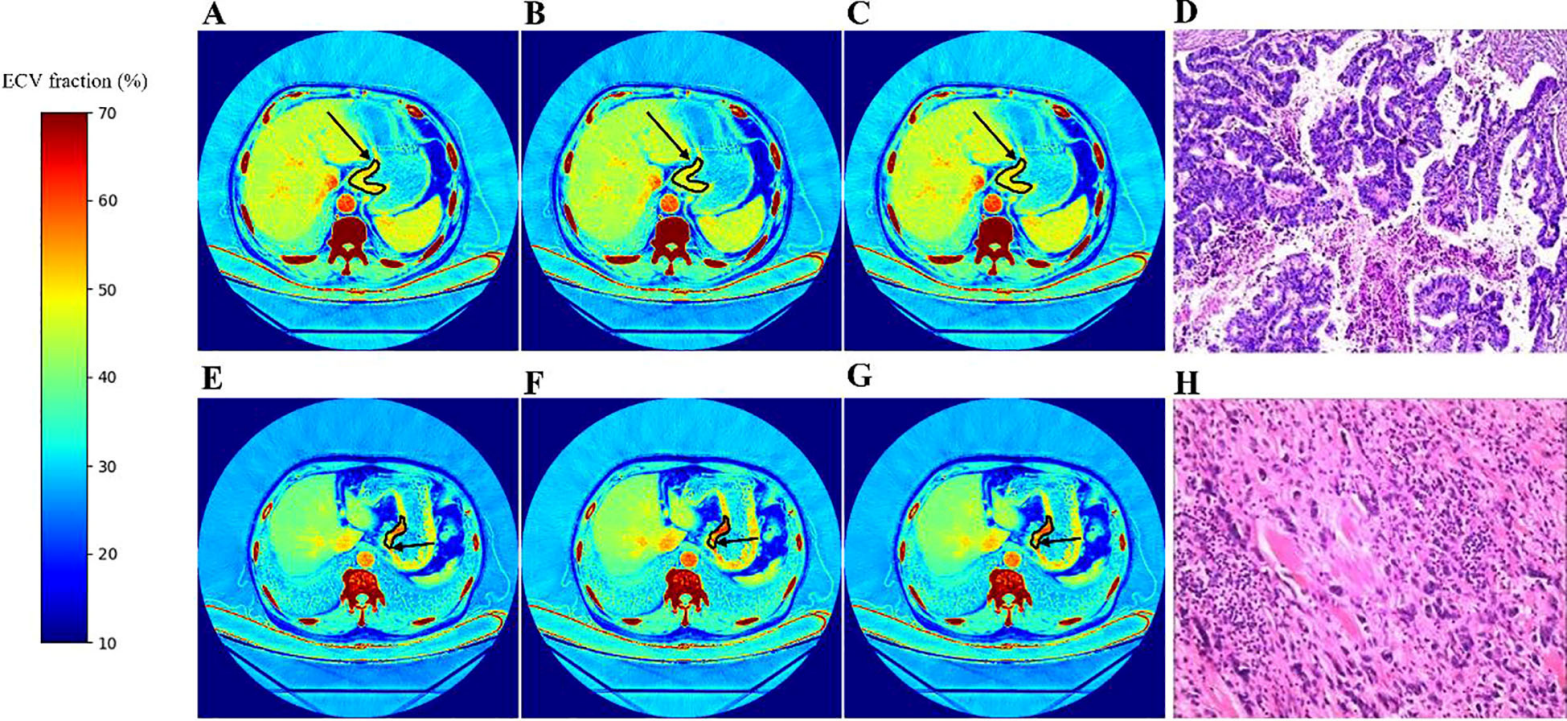
The results of synthetic ECV fractions. **(A-D)** corresponds to a 60-year-old patient with moderately differentiated gastric cancer in the low-grade group. **(E-H)** corresponds to a 76-year-old patient with poorly differentiated gastric cancer in the high-grade group. **(A, E)** are conventional ECV hot maps. **(B, F)** are ECV hot maps calculated by aortic CT attenuation. **(C, G)** are ECV hot maps calculated by inferior vena cava CT attenuation. **(D)**, **(H)** are pathological images (HE×100). The black arrows indicate the measurement points of the lesion ECV fractions, with their ECV fractions being: 0.405 **(A)**. 0.405 **(B)**. 0.432 **(C)**. 0.516 **(E)**. 0.542 **(F)**. and 0.533 **(G)**. respectively.

The derivation cohort consisted of 142 patients, including 85 males and 57 females, with an average age of 63(56,71) years. The validation cohort consisted of 62 patients, including 47 males and 15 females, with an average age of 64.210 ± 6.161 years, HCT of 40.300 (35.575,43.050) %, CT_A, V_ of 44.435 ± 4.913 HU, and CT_V, V_ of 43.288 ± 4.416 HU. Based on pathological confirmation, 23 individuals were in the low-grade group, 39 in the high-grade group, 44 with tubular adenocarcinoma, and 18 with mixed adenocarcinoma (tubular adenocarcinoma + low-adhesion carcinoma), as presented in **Table 1** and **Figure 3**. The CT attenuations or iodine concentrations measured by the two radiologists in the derivation cohort and the validation cohort were consistent. The ICC of the CT attenuation measurements in the derivation cohort was 0.85, the ICC of the CT attenuation measurements in the validation cohort was 0.82, and the ICC of the iodine concentration measurements was 0.79.

**Table 1:** Patient demographics.

Patient characteristics	Derivation cohort (n=142)	Validation cohort (n=62)	*P* value
Sex			0.028
Female	57	15	
Male	85	47	
Age (years)	63 (56, 71)	64.210 ± 6.161	0.539
HCT (%)	37.512 ± 6.081	40.300(35.575, 43.050)	0.133
CT_A_ (HU)	43.232 ± 4.858	44.435 ± 4.913	0.107
CT_V_ (HU)	42.683 ± 4.739	43.288 ± 4.416	0.393
BMI (kg/m^2^)	23.438 (21.484, 25.861)	23.380 ± 9.461	0.966
Pathological grade			
low-grade group	/	23	
high-grade group	/	39	
CTDIvol (mGy)	13.626 ± 0.639	13.530 ± 0.820 (unenhanced) 8.54 ± 0.000 (EP)	
DLP (mGy·cm)	740.779 ± 62.200	703.330 ± 73.186 (unenhanced) 448.282 ± 30.378 (EP)	
ED (mSv)	11.112 ± 0.933	10.550 ± 1.098 (unenhanced) 6.724 ± 0.456 (EP)	

HCT, hematocrit; CT_A,_ nonenhanced CT value of the abdominal aorta; CTv, nonenhanced CT value of the inferior vena cava. BMI, Body mass index;CTDIvol, Volume computed tomography dose index; DLP, Dose–length product; ED, Effective dose, ED = f * DLP, f=0.015; EP, equilibrium phase.

### 3.2 Calculation equation for synthetic HCT

The HCT of the 142 patients included in the derivation cohort was 37.512 ± 6.081%, with 43.232 ± 4.858 HU for CT_A_ and 42.683 ± 4.739 HU for CT_V_. The calculation equation of the synthetic HCT obtained from the derivation cohort was as follows (Equations 7, 8):

(7) $$HCT_{syn,A}\%=1.152\times CT_{A}-12.311$$

(8) $$HCT_{syn,V}\%=1.142\times CT_{V}-11.229$$

**Figure 4:**
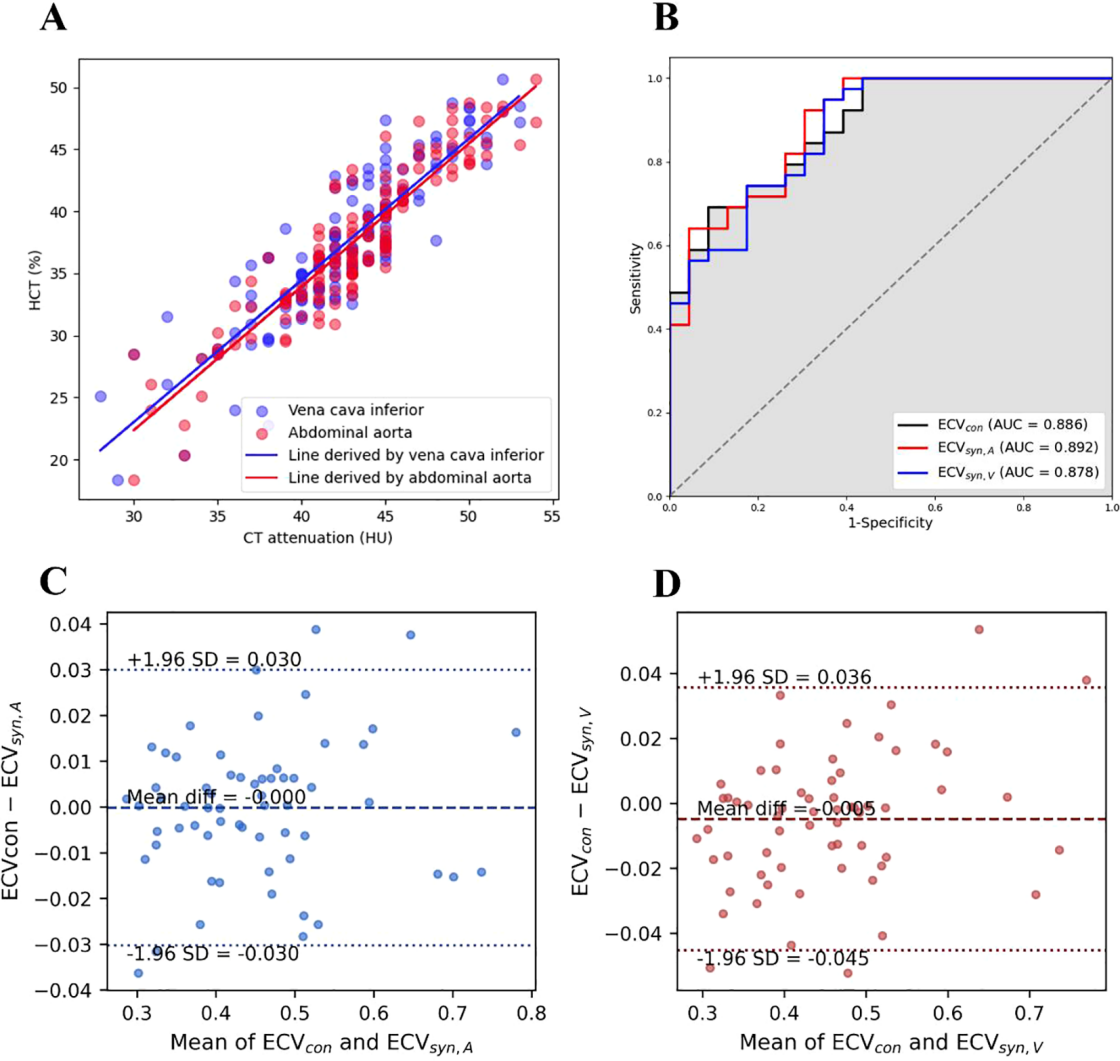
Summary of synthetic hematocrit and synthetic extracellular volume fraction analyses. **(A)** Linear regression graph of the CT attenuations of the abdominal aorta and inferior vena cava in the derived cohort with the HCT of blood. The acquired linear regression equations are HCT_syn, A_% = 1.152×CT_A_ - 12.311 and HCT_syn, V_% = 1.142×CT_V_ - 11.229. The R^2^s are 0.844 and 0.792, respectively. **(B)** The ROC curves of ECV_con_, ECV_syn, A_, and ECV_syn, V_ for differentiating gastric cancer pathological grade. ECV_con_, ECV_syn, A_, and ECV_syn, V_ exhibit favorable discriminatory efficacy for the pathological grading of gastric cancer, with AUC values of 0.886, 0.892, and 0.878, respectively. **(C, D)** Bland–Altman plots comparing synthetic and conventional ECV.

**Figure 4A** presents the effect of linear regression. The goodness of fit (R^2^) for the linear regression using the CT value of the abdominal aorta was 0.844. The R^2^ for the inferior vena cava was 0.792.

### 3.3 Synthetic of HCT and synthetic ECV

In the validation cohort, there were no statistically significant differences when conducting a pairwise comparison of the HCT_con_, HCT_syn, A_, and HCT_syn, V_ (all *P* > 0.05). A good correlation was observed between HCT_con_ and HCT_syn, A_ (r = 0.898, *P* < 0.01) as well as between HCT_con_ and HCT_syn, V_ (r = 0.826, *P* < 0.01). For the ECV_con_, ECV_syn, A_, and ECV_syn, V_, no statistically significant differences were found when conducting a pairwise comparison (all *P* > 0.05); a good correlation was found between ECV_con_ and ECV_syn, A_ (r = 0.990, *P* < 0.01) and between ECV_con_ and ECV_syn, V_ (r = 0.983, *P* < 0.01) (**Table 2**).

**Table 2:** Differences between synthetic and conventional HCT in the validation cohort, as well as between synthetic and conventional ECV.

Items	Conventional	Derived from		P1	P2	P3
		Abdominal aorta	Vena cava inferior			
HCT (%)	40.300(35.575, 43.050)	40.140(36.902, 42.230)	39.476(35.987, 42.068)	0.106	0.104	0.342
ECV	0.451 ± 0.111	0.451 ± 0.108	0.456 ± 0.1104	0.993	0.803	0.808

HCT, hematocrit; ECV, extracellular volume; P1, comparison between conventional values and values derived from the abdominal aorta; P2, comparison between conventional values and values derived from the vena cava inferior; P3, comparison between values derived from the abdominal aorta and vena cava inferior.

**Figures 4C, D** presents the Bland-Altman analysis between conventional ECV and synthetic ECV. The results indicated good consistency between ECV_con_ and ECV_syn, A_, with an average difference of <-0.001 (the lower limit of consistency was -0.030, the upper limit of consistency was 0.030, *P* < 0.01). Additionally, good consistency was observed between ECV_con_ and ECV _syn, V_, with an average difference of -0.005 (the lower limit of consistency was -0.045, the upper limit of consistency was 0.036, *P* < 0.01).

### 3.4 The discriminatory efficacy

The ECV_con_ in the high-grade group was 0.500 ± 0.103, the ECV_syn, A_ was 0.500 ± 0.101, and the ECV_syn, V_ was 0.502 ± 0.098. The ECV_con_ in the low-grade group was 0.367 ± 0.062, the ECV_syn, A_ was 0.369 ± 0.060, and the ECV_syn, V_ was 0.378 ± 0.060. The ECV values in the high-grade group were significantly greater than those in the low-grade group (all *P* < 0.001) (**Table 3**). Additionally, ECV_con_, ECV_syn, A_, and ECV_syn, V_ all had favorable discriminatory efficacy for the pathological grading of gastric cancer, with area under curve (AUC) values of 0.886, 0.892, and 0.878, respectively (**Figure 4B**). The DeLong test indicated that there was no statistically significant difference in the discriminatory efficacy of ECV_con_, ECV_syn, A_, and ECV_syn, V_ for the pathological grading of gastric cancer (**Table 4**).

**Table 3:** The difference in EVCs between high-grade and low-grade gastric cancer.

Group	ECV_con_	ECV_syn, A_	ECV_syn, V_
High-grade	0.500 ± 0.103	0.500 ± 0.101	0.502 ± 0.098
Low-grade	0.367 ± 0.062	0.369 ± 0.060	0.378 ± 0.060
*P*	<0.001	<0.001	<0.001

ECV_con_, conventional extracellular volume; ECV_syn, A_, Synthetic ECV calculated from synthetic HCT based on CT attenuation of the abdominal aorta; ECV_syn, V_, Synthetic ECV calculated from synthetic HCT based on CT attenuation of the inferior vena cava

**Table 4:** Discriminative efficacy of ECV_con_, ECV_syn, A_, and ECV_syn, V_ between gastric cancer pathological grades.

Item	ECV_con_	ECV_syn, A_	ECV_syn, V_
AUC	0.886	0.892	0.878
Delong’s test	*P1*	*P2*	*P3*
Value	0.631	0.603	0.298

AUC, area under curve; ECV_con_, conventional extracellular volume; ECV_syn, A_, Synthetic ECV calculated from synthetic HCT based on CT attenuation of the abdominal aorta; ECV_syn, V_, Synthetic ECV calculated from synthetic HCT based on CT attenuation of the inferior vena cava; P1, the Delong test P value between ECV_con_ and ECV_syn, A_; P2, the Delong test P value between ECV_con_ and ECV_syn, V_; P3, the Delong test P value between ECV_syn, A_ and ECV_syn, V_.

## 4 Discussion

This study shows that synthetic ECV is comparable to conventional, hematocrit-based ECV for grading gastric cancer, with no significant pairwise differences and strong correlations across metrics, and high discriminative performance (AUC ≈ 0.90) in distinguishing high-grade from low-grade tumors. These findings extend the concept of synthetic ECV beyond cardiovascular applications to the gastrointestinal domain, indicating that a blood-test-free approach can preserve diagnostic utility for gastric cancer grading while simplifying clinical workflow.

Several cardiac MRI and CT studies have demonstrated that synthetic HCT and synthetic ECV can reliably approximate conventional, blood-test-based ECV measurements, enabling quantitative assessment without the need for contemporaneous hematocrit sampling (14–17) These studies reported strong correlations and good agreement between synthetic and conventional ECV, supporting the feasibility of a blood-test–free workflow in routine clinical practice. Building on this foundation, our study extends the application of synthetic ECV from the cardiovascular system to the gastrointestinal domain, showing that synthetic ECV maintains equivalent performance to conventional ECV specifically for gastric cancer pathological grading. This organ-specific validation suggests that synthetic ECV may serve as a generalizable and reproducible quantitative imaging biomarker across different anatomical regions, not limited to myocardial fibrosis assessment. Additionally, we discovered that the correlation between synthetic and conventional ECV was greater than that between synthetic and conventional HCT, further supporting previous research data (18).

The extracellular volume fraction reflects the proportion of extracellular matrix (ECM) within the tumor microenvironment. Changes in ECM composition, vascular permeability, and stromal remodeling are known to accompany increasing tumor dedifferentiation (19, 20). Prior studies have shown that imaging-derived ECV correlates with histological markers of fibrosis, matrix expansion, and stromal infiltration in malignant tumors (21–25). Consistent with these biological features, our results demonstrated significantly higher ECV values in high-grade gastric cancers compared with low-grade tumors, supporting the concept that ECV can serve as a quantitative imaging surrogate of tumor aggressiveness.

Histopathological grading is a critical pathological parameter for evaluating tumor malignancy, based on the morphological characteristics, structural architecture, and degree of cellular differentiation of tumor cells. Different histological grades not only reflect variations in tumor biological behavior, but also directly guide clinical treatment decisions, prognostic assessment, and therapeutic evaluation, thus serving as a cornerstone in oncologic management. The fundamental basis of histopathological grading lies in the similarity between tumor cells and their tissue of origin, namely the degree of differentiation; a lower degree of differentiation corresponds to a higher degree of malignancy.

Previous studies have demonstrated that extracellular volume fraction (ECV) can serve as an imaging biomarker reflecting the expansion of the tumor extracellular matrix and remodeling of the tumor microenvironment. Similar to the research conclusion of Li et al.(11), our study demonstrated that ECV in high-grade gastric adenocarcinoma was significantly higher than that in low-grade tumors (P < 0.01), further supporting the feasibility and clinical potential of ECV as a noninvasive imaging biomarker for preoperative pathological grading in gastric cancer.

Both abdominal aorta–based and inferior vena cava–based synthetic ECV measurements showed good agreement with conventional ECV in our study, consistent with prior reports (8). However, the agreement between ECV_syn, A_ and ECV_con_ was slightly closer than that between ECV_syn, V_ and ECV_con_, which may be related to the greater stability and less compressibility of the abdominal aorta compared with the inferior vena cava during imaging. Since the inferior vena cava is more prone to deformation from respiration and abdominal pressure, measurements derived from the aorta may provide improved reproducibility in routine practice. Therefore, although both approaches are feasible, aortic ROI-based synthetic ECV may be preferred for clinical implementation.

From a clinical perspective, the key value of synthetic ECV lies not in outperforming conventional ECV, but in its practical feasibility and accessibility, workflow integration, and translational potential. Conventional ECV calculation requires contemporaneous hematocrit sampling, which may not always be available in outpatient, emergency, or retrospective imaging settings. In contrast, synthetic ECV can be derived directly from non-enhanced CT data, allowing immediate ECV assessment even when laboratory measurements are missing or delayed. This approach has been validated across cardiac and oncologic imaging studies: Treibel et al. (7)demonstrated that synthetic ECV derived from cardiac CT strongly correlates with conventional ECV (R² = 0.96) and histologic collagen volume fraction, confirming its biological validity; Kim et al. (17) further extended the method using dual-energy CT virtual unenhanced images, achieving automated ECV mapping during routine cardiac imaging; and Minamiguchi et al. (26) recently showed that synthetic tumor ECV on contrast-enhanced CT predicted both overall and recurrence-free survival in colorectal liver metastasis patients, highlighting its role as a prognostic imaging biomarker. In this context, our findings indicate that synthetic ECV in gastric cancer imaging provides a noninvasive, reproducible, and readily accessible quantitative surrogate for tissue microenvironment evaluation. By eliminating dependence on hematocrit sampling and leveraging routinely acquired CT data, synthetic ECV offers greater workflow simplicity, inter-institutional consistency, and scalability for large-scale retrospective analyses. Thus, rather than being a replacement for conventional ECV, synthetic ECV represents a clinically pragmatic and methodologically innovative alternative—bridging the gap between quantitative imaging and real-world clinical application.

Furthermore, the traditional calculation formula based on CT attenuations (2, 5, 7, 9) for ECV is ECV = (1 - Hct) × (ΔHU _Tumor_/ΔHU _Blood Pool_); ΔHU _Tumor_ and ΔHU _Blood Pool_ represent the differences between the CT attenuations of the solid part of the tumor and the blood pool at the lesion level during the equilibrium phase and the nonenhanced CT, respectively. The calculation formula for iodine maps is ECV = (1 - Hct) × (IC_lesion_/IC_aorta_); (IC_lesion_ and IC_aorta_ represent the iodine concentrations within the tissue and the blood pool during the equilibrium period, respectively). In this study, iodine concentration was used to calculate ECV. Compared with the method of measuring nonenhanced CT attenuations, measuring iodine concentration can reduce the radiation dose and eliminate registration errors (9, 10). In particular, as the stomach is an organ capable of peristalsis, DECT can offer a more accurate assessment of ECV for gastric cancer (27).

Our research has several limitations. The primary constraint is the relatively small sample size. All the examinations were conducted at a single center, and there was no well-established external validation. Second, there are no histopathological reference standards, such as the collagen volume fraction. Finally, only one DECT scanners were utilized for estimating the synthetic hematocrit and calculating the ECV. It is currently unknown whether these results can be replicated on other scanning protocols and DECT platforms. It is important to acknowledge that the quantitative formulas proposed in this study were derived based on a specific scanner platform and reconstruction protocol. Variations in tube voltage, detector materials, spectral filtration, and image-domain post-processing may introduce differences in measured attenuation values, thereby affecting the calculation of synthetic HCT and potentially limiting the direct applicability of the proposed formulas to other imaging systems. When implementing this method on new equipment or under different protocols, it is necessary to replicate the derivation process using a representative cohort within the new environment to establish a formula tailored to that specific setting. Consequently, further research-including multicenter external validation and protocol harmonization-is warranted to confirm the robustness and reproducibility of our approach across different vendors and acquisition conditions. Another drawback of this approach is that the synthetic ECV calculation has the potential to misclassify the ECV of individual patients. Treibel et al. (7) reported that the variation at the level of each patient can be as high as 8%. This deficiency might also be similar in synthetic ECV-MRI calculations. Chen et al. (8) demonstrated that even when the synthetic ECV was calculated using scanner-specific models, compared with the ECV calculated using blood HCT levels, the ECV could be underestimated or overestimated by 4%, and this situation could also occur with CT.

## 5 Conclusions

In summary, the application of the synthetic ECV method in gastric cancer is feasible. The calculation of hematocrit using the CT attenuation coefficient of the unenhanced scan is nearly the same as that of the conventional method, avoiding the inconvenience of blood collection. Although there was no significant difference between the synthetic ECV obtained at the abdominal aorta level and the inferior vena cava level, the error between the synthetic ECV calculated at the abdominal aorta level and the conventional ECV was smaller. In conclusion, the synthetic ECV calculated based on synthetic HCT is comparable to the conventional ECV based on serum HCT, and both have good predictive value for the pathological grading of gastric cancer.

## Data Availability

The raw data supporting the conclusions of this article will be made available by the authors, without undue reservation.
